# Biofilm Formation Plays a Role in the Formation of Multidrug-Resistant *Escherichia coli* Toward Nutrients in Microcosm Experiments

**DOI:** 10.3389/fmicb.2018.00367

**Published:** 2018-03-02

**Authors:** Xiu P. Chen, Liaqat Ali, Li-Yun Wu, Can Liu, Chen X. Gang, Qi F. Huang, Jing H. Ruan, Song Y. Bao, Yun P. Rao, DaoJin Yu

**Affiliations:** ^1^Fujian Key Laboratory of Traditional Chinese Veterinary Medicine and Animal Health, Fujian Agriculture and Forestry University, Fuzhou, China; ^2^Department of Biosciences, Faculty of Sciences, COMSATS Institute of Information Technology, Islamabad, Pakistan

**Keywords:** microcosm, nitrogen, phosphorus, *Escherichia coli*, antibiotic resistance, biofilm, biofilm-forming related genes, no observed effect concentration (NOEC)

## Abstract

In this study, microcosms were established to determine the effect of nitrogen (N) and phosphorus (P) on the multidrug resistance and biofilm-forming abilities of *Escherichia coli*. The expression of biofilm-formation-related genes was detected to establish correlations between genotype and phenotype. Different concentrations of N and P were added to make one control group and four treatment groups. The glass tube method was used to determine biofilm-forming capabilities. Real-time PCR was used to detect the mRNA abundance of six biofilm-formation-related genes in *E*. *coli*. No resistant strains were isolated from the control group; meanwhile, multidrug resistance rates were high in the treatment groups. Expression of the biofilm-associated genes *luxS, flhD, fliA, motA*, and *fimH* was detected in all treatment groups; however, there was no expression of *mqsR*. The expression of *luxS, flhD, fliA, motA*, and *fimH* significantly correlated with the concentration of N and P, as well as with the appearance and duration of multidrug resistance in different groups. Overall, the results of this study suggest that biofilm-forming ability plays a key role in the formation of multidrug resistance in *E*. *coli* after the addition of N and P to a microcosm.

## Introduction

Diverse aquatic microorganisms including *Escherichia coli* population stick to each other to form a colonizing surface which leads to the formation of biofilm (Dang and Lovell, 2016). In these associations, the microbes are exposed to diverse environmental conditions such as nutrient availability, temperature, pH, and so on. One of the main properties of biofilm development is to promote resistance to antibiotics compared with planktonic cells (Soto, 2013). Furthermore, biofilms protect microbes against environmental stresses and result in increased resistance to antimicrobial treatments and disinfectants (Sanchez-Vizuete et al., 2015). The reduced sensitivity of biofilms to antibiotics is a critical problem in the chronic infection treatments. Approximately 65% of bacterial infections are linked with biofilms (Romling and Balsalobre, 2012). Furthermore, biofilm are 100–1,000 times more resistant to antibiotics than planktonic bacteria (Ito et al., 2009).

Biofilm resistance is one of the main modes of transmission of drug resistance in *E. coli*. It is also the way that most biofilm forming bacteria survive in hostile environments. In drinking water, the biofilm development is dependent on the physiological state of *E*. *coli* rather than anaerobic conditions (Farkas et al., 2016). Bjergbæk et al. (2006) found that minimal and complex media may affect *E*. *coli* biofilm development in aerobic condition. Furthermore, this bacterium is also capable to develop biofilm in anaerobic conditions. Higher phosphorus (P) concentrations in drinking water promote biofilm and prolonged the survival of *E*. *coli* (Juhna et al., 2007). Cerca and Jefferson (2008) found that while the expression of *pga* (a gene operon of *E*. *coli*) and polysaccharide synthesis were induced by ethanol, NaCl, and glucose, however, biofilm formation was only induced by glucose. At a high glucose concentration (1 g⋅L^-1^), the extreme biofilm volume was achieved after 24 h; similar values were obtained in incubators with orbital diameters of both 50 and 25 mm. At a low glucose concentration (0.5 g⋅L^-1^), lower biofilm volumes were obtained (Moreira et al., 2013).

Biofilm development within aquatic environment depends on the chemical and physical characteristics of the water and sediments. In the underlying sediments, nutrient, dissolved organic carbon, carbon dioxide, and oxygen concentrations can vary in spatial and temporal regions with respect to temperature, pH, diurnal, and annual changes that lead to horizontal and vertical stratifications of biofilm development. In the sediment, nutrients such as nitrogen (N) and phosphorus (P) play important role in the biofilm development (Ahmad et al., 2017; Shrestha and Afsar, 2017). Previous studies have reported that cornmeal; N and P induced fecal colonization and antibiotic resistance in mesocosm experiments. Mesocosm constructions are useful tools because these approaches allow researchers to simulate realistic environmental conditions, replicates of different treatments, and extended periods of experimentation (Taub, 1997). In recent years, controlled conditions have been used to evaluate the bacterial biofilm development in marine and freshwater, using mesocosm and characterized by taxonomy, microscopy, gravimetry, chemical analysis and molecular biology (Zippel et al., 2007; Ali et al., 2016).

To the best of our knowledge, previous studies have not examined the direct relationship between nutritional factors and biofilm formation and drug resistance, nor dose-effect and time-effect associations. Biofilm formation-related genes play different roles in biofilm formation; it is necessary to determine the expression levels of these genes, as well as the relationship between their expression levels and the level of nutrition and drug resistance.

We have previously studied the impacts on the drug resistance of *Enterococcus* spp. and *E*. *coli* of adding cornmeal, glucose, nitrogen (N), and phosphorus (P) nutrients to mesocosms (Yu et al., 2009, 2012; Xiao et al., 2015; Ali et al., 2016; Zhang et al., 2016). Yu et al. (2009) established mesocosm experiments to study the effects of feed residue on the acquired antibiotic resistance of bacteria in sediment. Furthermore, Yu et al. (2012) examined the antibiotic resistance in a mesocosm containing different concentrations of cornmeal. In the presence of several environmental factors in aquatic environments, biofilm development is one of the best survival strategies for microbes. Previous studies have often focused on bacterial resistance, while little is known about biofilm development in aquatic environments. In this study, the effect of N and P on the multidrug resistance and biofilm-forming abilities of *E*. *coli* were examined, as well as the correlations between the expression of biofilm-formation-related genes and different stages of biofilm formation. This provides a deeper understanding of the mechanism of bacterial resistance caused by nutritional factors, improves awareness of environmental pollution, and provides a theoretical basis for improved control of bacterial resistance.

## Materials and Methods

### Materials

#### Microcosm

Sediment: soil without antibiotics was collected from Fuzhou Forest Park, Fujian Province, China. Water: moderately filtered water (single distilled water) was exposed to sunlight for 1 day. Plastic bucket: a white transparent storage box with a volume of 12 L. Tents: 3 m × 3 m × 2 m, surrounded by white transparent plastic film, with top covering of a plastic film. The microcosms were arranged in a randomized complete block design in five blocks. Each block represents different treatment (three replicate microcosms per treatment).

#### Standard Antibiotics

Ampicillin, cefazolin, cefotaxime, gentamicin, ciprofloxacin, tetracycline, and chloramphenicol were purchased from the China Pharmaceutical and Biological Products Inspection Institute.

#### Reagents

Premix *Taq*^TM^ (Takara *Taq*^TM^ Version 2.0), a PrimeScript RT reagent Kit with gDNA Eraser, SYBR^®^ Premix Ex *Taq*^TM^ (Tli RNaseH Plus), DL500 Marker, and 6 × Agarose Gel Loading buffer were purchased from Takara (Dalian, China). TAE buffer (5 ×) was purchased from Sangon Biotech Co., Ltd. (Shanghai, China).

#### Standard Strain

*Escherichia coli* (ATCC 25922) was purchased from the National Institute for the Control of Pharmaceutical and Biological Products (Beijing, China).

### Methods

#### Microcosm

Autoclaved soil (15 kg) was evenly distributed in 12 L white transparent pails and mixed with 10 L of distilled water and the appropriate standard dilution of *E*. *coli* ATCC 25922. The water level of each pail was marked and maintained at a constant volume. The transparent buckets were placed in plastic greenhouses for 30 days, with about 10 cm between buckets.

#### Sample Collection

Bacterial samples were collected from the microcosm sediments on days 1, 8, 15, 22, 29, 36, 54, and 85 as described previously (Yu et al., 2012). These samples were collected from four random locations of each microcosm (five treatments and three replicates) for further analyses. In order to avoid overlapping, the samples were collected from consistently dispersed points of the entire microcosm. The total nitrogen (TN) and total phosphorus (TP) levels were measured during incubation. Once the system was set up, the water-sediment interface was sampled (0 day). Then, sterilized N (NH_4_NO_3_) and P (NaH_2_PO_4_) were added at the specified concentrations. Samples were taken at 1, 8, 15, 22, 29, 36, 54, and 85 days. There was one control group and four treatment groups; each group was run in triplicate. Based on preliminary tests (data not shown), the added concentrations of TN and TP in the microcosm were lowered. The final nutrient concentrations of all treatment groups ranged from moderately eutrophic to hypertrophic (**Table 1**).

**Table 1:** Total N and total P treatments in the microcosm.

	Treatment groups				
	Blank group	Lowest-dose group	Low-dose group	Medium-dose group	High-dose group
Bucket number	a,b	1, 2	3, 4	5, 6	7, 8
TN (mg⋅L^-1^)	0.65	1.5	2.5	3.5	4.5
TP (mg⋅L^-1^)	0.015	0.15	0.25	0.35	0.45

*The concentrations of TN and TP stated are added to the microcosms under five different TN and TP regimes. The blank control is at the medium nutrition level. The low- and medium-dose groups are at the middle eutrophication level, and the high-dose group is at the high eutrophication level.*

#### *Escherichia coli* Sensitivity Test

Colonies were suspended in Mueller-Hinton Broth (MHB) for 6 h and quantified using an ultraviolet Spectrophotometer (Thermo Fisher Scientific Inc., Wilmington, DE, United States). The cultures were then diluted with sterilized MHB broth (about 10 times to achieve the absorbance value between 0.08 and0.10). Broth dilution technique was carried out to determine resistance to the antimicrobials (**Table 2**).

**Table 2:** Standard of equivalent minimal inhibitory concentration (MIC) breakpoints for *Escherichia coli*.

Antibiotic	MIC (μg⋅mL^-1^)		
	S	I	R
Ampicillin	≤8	16	≥32
Cefazolin	≤1	2	≥4
Cefotaxime	≤1	2	≥4
Gentamicin	≤4	8	≥16
Ciprofloxacin	≤1	2	≥4
Tetracycline	≤4	8	≥16
Chloramphenicol	≤8	16	≥32

*S, sensitive; I, intermediate; R, resistant.*

Antibiotic resistance testing was performed according to the broth microdilution method (Clinical and Laboratory Standards Institute [CLSI], 2015). Strains with an MIC less than or equal to the break points were considered susceptible; those higher than the break points were considered resistant. Susceptibility test results were interpreted using Clinical and Laboratory Standards Institute [CLSI] (2006) breakpoint criteria (**Table 2**).

#### Glass Tube Test

A tube containing 1 mL of liquid medium was inoculated with 10 μL of *E*. *coli* and incubated at 37°C for 17 h. Then, the medium was removed and the cells were stained with 1.5 mL of 1% crystal violet at 18–20°C (room temperature) for 30 min. The crystal violet staining solution was removed by washing with clear water until colorless water was obtained; then, the cells were dried at room temperature. Visual observation was performed to observe the junction of the liquid level and color of the glass tube wall. Pictures were taken to record the results.

#### Real-Time PCR

Total RNA was extracted from 45 samples using TRIzol method (Invitrogen) and cDNA was obtained by reverse transcription kit (Takara Dalian, China). Real-time PCR was performed according to the conditions described previously (Maeda et al., 2003). Seven sets of PCR primers (**Table 3**) were used (synthesized by Sangon Biotech). To ensure primer specificity, agarose gels with the PCR product were run and product melting was assessed at the end of the reaction to verify the specificity of the reaction (**Supplementary Figure S1**). Standards for all seven genes (*gapA, luxS, mqsR, flhD, fliA, motA*, and *fimH)* were prepared and were serially diluted over five or six orders of magnitude to generate a standard curve (**Supplementary Figure S2**). The PCR conditions were: pre-denaturation at 95°C for 2 min, then 39 cycles of denaturation at 95°C for 15 s, annealing at 62°C for 15 s, and extension at 72°C for 20 s. After the cycling program, a melting curve was generated by cooling the samples to 65°C for 2 min and then slowly increasing the temperature to 95°C with a slope of 0.2°C/s while measuring fluorescence continuously.

**Table 3:** List of primers used in this study.

Gene	Length	Primer	Sequence	Reference
*LuxS*	116 bp	*luxS-F*	5′-TGCCACACTGGTAGACGTTC-3′	This study
		*luxS-R*	5′-TGATTGGTACGCCAGATGAG-3′	
*MqsR*	105 bp	*mqsR-F*	5′-ACGCACACCACATACACGTT-3′	This study
		*mqsR-R*	5′-TCCAAACCTAACTCATCTGCAT-3′	
*FlhD*	135 bp	*flhD-F*	5′-ATCGTCTGGTGGCTGTCAA-3′	This study
		*flhD-R*	5′-GTCCGCTATGTTTCGTCTCG-3′	
*FliA*	112 bp	*fliA-F*	5′-GCTGGCTGTTATTGGTGTCG-3′	This study
		*fliA-R*	5′-CAACTGGAGCAGGAACTTGG-3′	
*MotA*	120 bp	*motA-F*	5′-CTTCCTCGGTTGTCGTCTGT-3′	This study
		*motA-R*	5′-CTATCGCCGTTGAGTTTGGT-3′	
*FimH*	127 bp	*fimH-F*	5′-GCGATGATTTCCAGTTTGTG-3′	This study
		*fimH-R*	5′-ATTGGCACTGAACCAGGGTA-3′	
*GapA*	104 bp	*gapA-F*	5′-GAAATGGGACGAAGTTGGTG-3′	Congrui et al., 2013
		*gapA-R*	5′-AACCACTTTCTTCGCACCAG-3′	

*The primers for the target genes were designed using the NCBI primer designing tool.*

### Statistical Analysis

Quantification cycle (Cq) values were obtained after completion of the fluorescence quantitative PCR reaction. The relative abundance of the target gene in each sample and calibrator (blank group) was calculated using the Cq value of the internal reference gene *gapA* as a control and the relative expression level of the target gene = 2^-ΔΔCq^ (where ΔCq = Cq_(target gene)_ - Cq_(reference gene)_, and ΔΔCq = ΔCq_(test)_ - ΔCq_(calibrator)_) as described previously (Livak and Schmittgen, 2001). Statistical analysis was performed using SPSS software (version 10.0).

## Results

### Multidrug Resistance in *E*. *coli*

In the control group, no resistant *E*. *coli* strains were obtained (**Table 4**). The multidrug resistance rate was highest in the medium N and P dose group; the rate was second highest in the high-dose group. The resistance rate was lowest in the lowest- and low-dose groups. After 1 day, high multidrug resistance rates were found in the lowest, medium, and high dose groups. In the medium dose group, there were multiple multidrug-resistant *E*. *coli* strains isolated on selected days after the addition of N and P. In all treatment groups, there were high levels of multidrug-resistant strains from day 29 to the end of the experiment.

**Table 4:** Multidrug resistance (%) of *Escherichia coli* in different dose groups.

Sampling time	Blank group	Lowest-dose group	Low-dose group	Medium-dose group	High-dose group
0 day	0%	0%	0%	0%	0%
1 day	0%	60%	0%	50%	57.14%
8 days	0%	69.23%	0%	81.82%	0%
15 days	0%	0%	50%	50%	0%
22 days	0%	0%	66.67%	100%	81.82%
29 days	0%	60%	57.14%	100%	100%
36 days	0%	100%	100%	83.33%	100%
54 days	0%	100%	100%	100%	100%
85 days	0%	100%	100%	100%	100%

*Blank group; total nitrogen (TN) amended with NH_*4*_NO_*3*_ to maintain the N concentration at 0.65 mg L^-*1*^ and total phosphorus (TP) amended with KH_*2*_PO_*4*_ to maintain the P concentration at 0.015 mg L^-*1*^, (ii) Lowest-dose group; addition of 1.5 mg L^-*1*^ N and 0.15 mg L^-*1*^ P (iii) Low-dose group; addition of 2.5 mg L^-*1*^ N and 0.25 mg L^-*1*^ P (iv) Medium-dose group; addition of 3.5 mg L^-*1*^ N and 0.35 mg L^-*1*^ P and (v) High-dose group; addition of 4.5 mg L^-*1*^ N and.45 mg L^-*1*^ P.*

### Biofilm Formation of *E*. *coli* in Different Dose Groups Detected by Glass Tube Method

**FIGURE 1:**
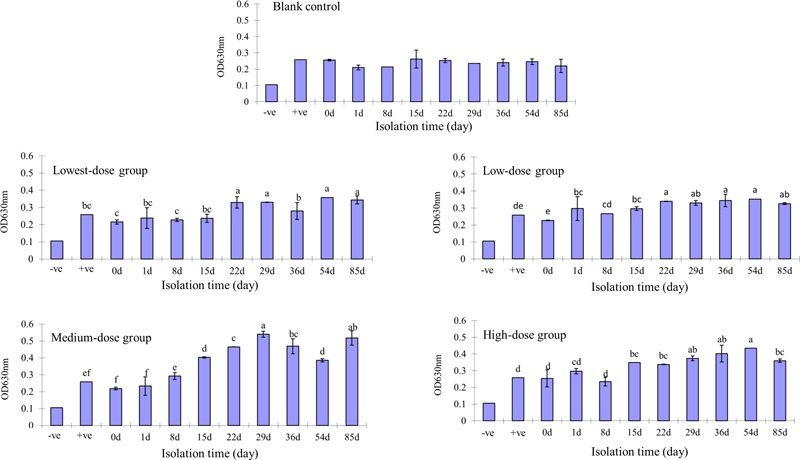
*Escherichia coli* biofilm developed by different-dose groups at different time points. Different lowercase letters indicate significant differences (*P* < 0.05); marked with the same lowercase letters indicate no significant difference (*P* > 0.05).

**FIGURE 2:**
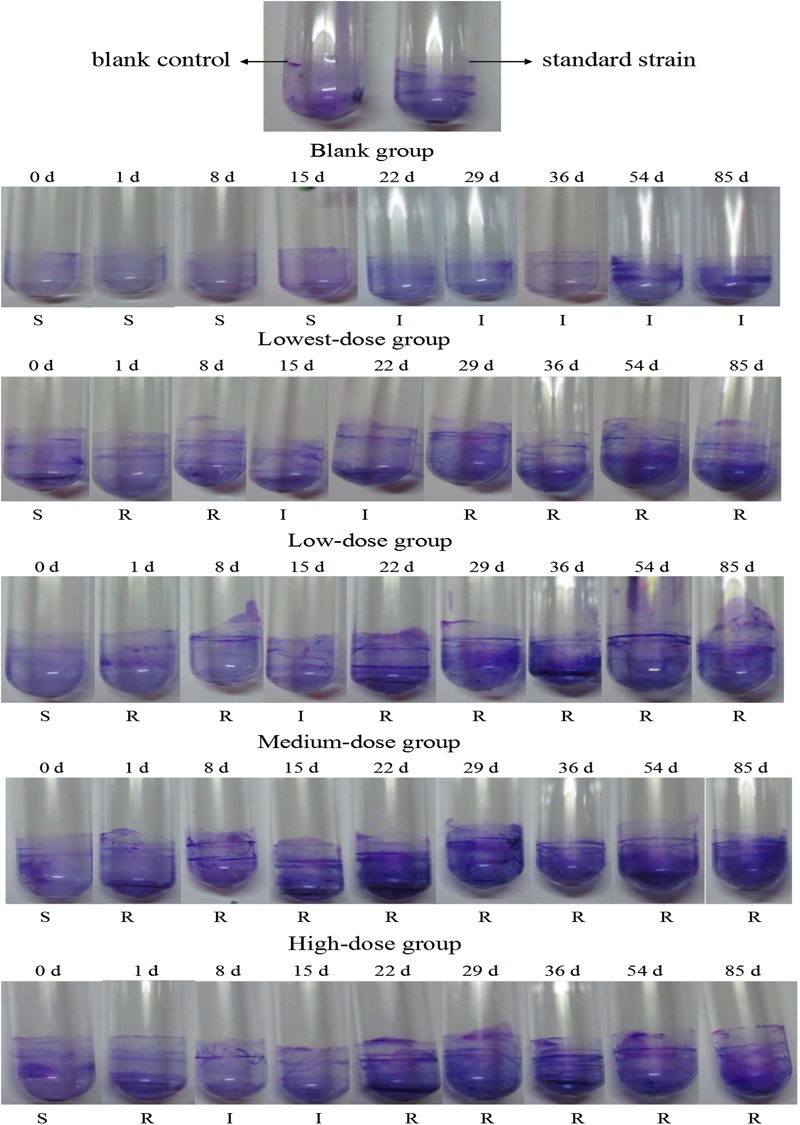
*Escherichia coli* biofilm developed by different-dose groups in test tubes. R, resistant; I, intermediate; S, sensitive.

There was no biofilm formation in the control group or at day 0 in any of the treatment groups. After adding N and P nutrients, biofilm formation in the medium-dose group was the highest, followed by the high-dose and two low-dose groups (**Figure 1**). The biofilm that formed on the glass tube of the middle-dose group was visually apparent from the 1st day onward; meanwhile, the biofilm in the other groups was visually apparent from the 29th day onward. There was a positive correlation between the multidrug resistance rate and biofilm formation (**Figure 2**).

### Results of Real-Time PCR

There was no *mqsR* gene expression observed throughout the study (samples collected on day 0 as controls). Five genes (i.e., the self-inducible gene *luxS*, motif *flhD, fliA, motA*, and *fimH*) were not expressed in the control group.

The gene expression level and duration trends of *flhD, fliA, motA*, and *fimH* in different treatment groups were consistent. The order of duration and expression level from highest to lowest was: the medium-dose group, the high-dose group, the low-dose group, and the lowest-dose group. The expression levels of the genes peaked after 8 days in the medium-dose group and high-dose group, and reached minima at the 15th and the 22nd day, respectively. The expression levels of the genes in the low-dose group and lowest-dose group peaked after 15 days, and reached minima at the 29th and 36th day, respectively. In the lowest-, low-, and medium-dose groups, *fliA* had the highest expression level, followed by *motA, fimH*, and *flhD*. The overall expression levels of the four genes were similar in the high-dose group.

**FIGURE 3:**
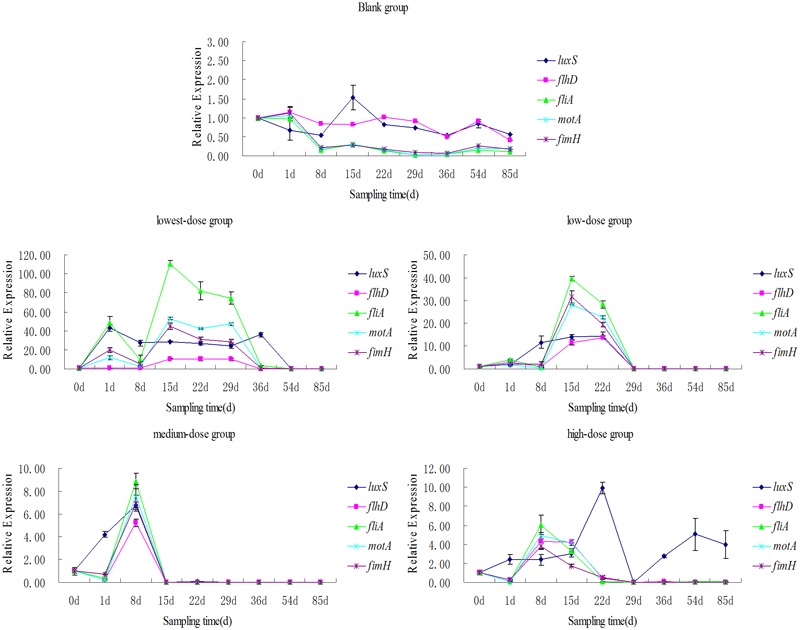
Relative expressions level of *luxS, flhD, fliA, mot*A, and *fimH* genes.

The *luxS* expression levels were similar in all groups except the high-dose group (**Figure 3**) and the hierarchy of *luxS* expression duration and level in the other three groups was the same as for the other genes tested. We found that *luxS* began to be expressed in the medium- and high-dose groups after 1 and 8 days, respectively; the expression level was significantly higher than that of the other four genes. Meanwhile, *luxS* was expressed on the 1st day in the lowest-dose group at a higher level than all of the other genes except *fliA*.

## Discussion

### Response of Drug Resistance to Environment

Thevenon et al. (2012) investigated multiple antibiotic resistance (MAR) of the fecal indicator bacteria (FIB) *E*. *coli* and *Enterococcus* spp. from sediments of Lake Geneva (Switzerland) over multiple decades. Their study showed that after 1970 sediments had deposited much higher levels of FIB with MAR than those before 1970. This is due to the fact that, after 1970, eutrophication greatly increased around Lake Geneva. Thus, the trophic state of the lake and municipal wastewater treatment plants had substantial effects on FIB and on the distribution of multidrug resistance genes in the sediments. In addition, certain areas of the bay constitute a reservoir of FIB and FIB with MAR in the contaminated sediments. Therefore, the suspension of FIB and FIB with MAR can impact health risks and water quality during recreational activities.

Our previous studies also found that FIB from microcosms quickly acquired resistance when subjected to different concentrations of cornmeal, glucose, or N and P (Yu et al., 2009, 2012). Here, the concentrations of N and P were lowered; however, they were still equivalent to a medium eutrophication level (the N and P levels in the control group were equivalent to a mesotrophic level). We have found that even small additions of any type of nutrient salt to a microcosm can alter the resistance characteristics of FIB (Ali et al., 2016; Zhang et al., 2016). The resistance mechanism(s) of FIB seem to be sensitive to environmental changes, and we believe that these resistance mechanisms play a key role in bacterial self-defense systems in changing conditions, and are even more important than antibiotics themselves (Rochelle-Newall et al., 2015). Thus, it would be of great interest to develop a new system capable of detecting the no observed effect concentration (NOEC) of glucose or N and P on the formation of resistance in FIB. Furthermore, these NOECs will be critical in evaluating the threat of FIB resistance in water sources.

### Relationship Between Biofilm-Forming Genes and MAR

Real-time fluorescent quantitative PCR showed that *E*. *coli mqsR* was not expressed (**Figure 3**). Although studies have shown that *mqsR* can regulates the motility master regulon *flhD* and play an important role in biofilm development (González Barrios et al., 2006), our results show that the formation of *E*. *coli* biofilms was not related to *mqsR* expression following the addition of low levels of N and P.

Quantitative fluorescence PCR showed that expression levels of *luxS, flhD, fliA, motA*, and *fimH*, genes involved in biofilm formation, increased to a certain extent in each treatment group (**Figure 3**). This provides further confirmation that *E*. *coli* uses biofilm formation to adapt to changes in the external environment, in this case, in response to the addition of low levels of N and P.

Niu et al. (2013) proved that *luxS* influences *E*. *coli* biofilm development independently of autoinducer-2 and can assist in adaptation to varied conditions. In our study, the expression of *luxS* was induced rapidly in response to the addition of nutrient salts; it was detected in the medium- and high-concentration groups on day one. Pratt and Kolter (1998) used *E*. *coli* as a model organism to examine the initiation of biofilm development. Their study showed that motility is critical for normal biofilm development. Furthermore, it has been presented that type I pili (harboring the *fimH* gene) is required for initial surface adhesion (Pratt and Kolter, 1998). Niu et al. (2013) also showed that fimbriae may promote surface colonization and other aspects of initial biofilm development by *E*. *coli*.

Based on the role of pili and kinetic (or motility) genes in biofilm formation, as well as the results of this study, we infer that the lack of *flhD, fliA, motA*, and *fimH* expression suggests that the mature biofilm has begun to form. After completion of the adhesion and microcolony formation stages, in which the flagella and pili play an important role, the bacteria begin to gradually enter the mature biofilm stage (Berne et al., 2015). The shorter the duration of gene expression and maintenance of these four genes, the earlier *E*. *coli* enter the mature biofilm forming stage.

We showed that the expression of the four genes was maintained for the shortest time in the medium-dose group, followed by the high-dose group, the lowest-dose group, and the low-dose group. Thus, the medium-dose group was the earliest to form a mature biofilm; this agrees with previous analyses of biofilm formation abilities (Abberton et al., 2016). In addition, judging from the overall gene expression levels, gene expression is positively correlated over time, indicating that the medium-dose group was the best-adapted to the external environment. It formed a mature and stable biofilm in the shortest time and at the lowest gene expression levels, which ensured the survival of the bacteria in a changing environment.

### The Expression Level of Biofilm Forming Genes and Their Relationship

The expression levels and trends of the three motor genes and pilus were consistent (**Figure 3**). The expression level of *fliA* was the highest, followed by *motA, fimH*, and *flhD*. In *E*. *coli, flg, flh*, and *fli* are the flagellum biosynthetic genes, and *mot* are motor genes (Chen and Helmann, 1992). The *fimH* adhesin is the tip protein of *E*. *coli* type I fimbriae (Lee et al., 2005). At the top of the hierarchy is the *flhD* master operon, which is consists of the *flhD* and *flhC* genes: expression of this regulon is essential for the expression of all of the remaining genes (**Supplementary Figure S3**). Class II operons consist of operons under direct control of the master operon. The genes contained in these operons encode a flagellum-specific sigma factor (σ^28^ or FliA) that is required for the transcription of class III operons, many structural components assembled in the early and middle stages of flagella synthesis, and some proteins of unknown function. Class IIIa regulons are under the dual control of *fliA* and *flhD/flhC*; meanwhile, class IIIb operons (such as *motA*) are under the control of *fliA* alone (Liu and Matsumura, 1994). Claret et al. (2007) showed that sigma factor FliA, transcriptional activator FlhD_2_C_2_, and flagella regulators are involved in the coordination of flagella and type I pilus synthesis. FliA is also a key regulatory component linking synthesis of the flagella and type I pili; its effect on type I pili is mediated via a c-di-GMP-dependent signaling pathway. Additionally, *fliA*, the key gene connecting flagella and pili, had the highest overall expression in our experiments, followed by *motA*, the motor gene (Belas, 2014). This is important since motility plays a critical role in surface contact and biofilm expansion during the initial stages of biofilm formation.

## Author Contributions

XC, LA, JR, L-YW, CL, QH, and DY conceived and designed the study. XC and LA performed the experiments. XC, LA, CG, SB, and YR analyzed the data and wrote the paper. All authors contributed to the editing of the manuscript.

## Conflict of Interest Statement

The authors declare that the research was conducted in the absence of any commercial or financial relationships that could be construed as a potential conflict of interest.

## Supplementary Material

FIGURE S1 **(A)** Electrophoresis result of six targeted genes “M” is DL500 marker; 1, 2, 3, 4, 5 and 6 is *luxS, mqsR, flhD, fliA, motA*, and *fimH* PCR products, respectively. **(B)** Melting peak of *luxS, mqsR, flhD, fliA, motA*, and *fimH* gene.

FIGURE S2 Standard curve of **(A)** *gapA*, **(B)** *luxS*, **(C)** *mqsR*, **(D)** *flhD*, **(E)** *fliA*, **(F)** *motA*, and **(G)** *fimH* gene.

FIGURE S3 The relationships between biofilm-forming genes are used with permission of (Liu and Matsumura, 1994).

## References

[B1] Abberton C. L. Bereschenko L. van der Wielen P. W. J. J. Smith C. J. (2016). Survival, biofilm formation, and growth potential of environmental and enteric *Escherichia coli* strains in drinking water microcosms. *Appl. Environ. Microbiol.* 82 5320–5331. 10.1128/AEM.01569-16 27342552 PMC4988207

[B2] Ahmad M. Liu S. Mahmood N. Mahmood A. Ali M. (2017). Effects of porous carrier size on biofilm development, microbial distribution and nitrogen removal in microaerobic bioreactors. *Bioresour. Technol.* 234 360–369. 10.1016/j.biortech.2017.03.076 28343055

[B3] Ali L. Wang Y. Q. Zhang J. Ajmal M. Xiao Z. Wu J. (2016). Nutrient-induced antibiotic resistance in *Enterococcus faecalis* in the eutrophic environment. *J. Glob. Antimicrob. Resist.* 7 78–83. 10.1016/j.jgar.2016.07.014 27685672

[B4] Belas R. (2014). Biofilms, flagella, and mechanosensing of surfaces by bacteria. *Trends Microbiol.* 22 517–527. 10.1016/j.tim.2014.05.002 24894628

[B5] Berne C. Ducret A. Hardy G. G. Brun Y. V. (2015). Adhesins involved in attachment to abiotic surfaces by Gram-negative bacteria. *Microbiol. Spectr.* 3 10.1128/microbiolspec.MB-0018-2015 26350310 PMC4566860

[B6] Bjergbæk L. A. Haagensen J. A. J. Reisner A. Molin S. Roslev P. (2006). Effect of oxygen and growth medium on in vitro biofilm formation by *Escherichia coli*. *Biofilms* 3 1–10. 10.1017/S1479050507002074

[B7] Cerca N. Jefferson K. K. (2008). Effect of growth conditions on poly-N-acetylglucosamine expression and biofilm formation in *Escherichia coli*. *FEMS Microbiol. Lett.* 283 36–41. 10.1111/j.1574-6968.2008.01142.x 18445167

[B8] Chen Y. F. Helmann J. D. (1992). Restoration of motility to an *Escherichia coli* fliA flagellar mutant by a *Bacillus subtilis* sigma factor. *Proc. Natl Acad. Sci. U.S.A.* 89 5123–5127. 10.1073/pnas.89.11.5123 1594620 PMC49241

[B9] Claret L. Miquel S. Vieille N. Ryjenkov D. A. Gomelsky M. Darfeuille-Michaud A. (2007). The flagellar sigma factor FliA regulates adhesion and invasion of Crohn disease-associated *Escherichia coli* via a cyclic dimeric GMP-dependent pathway. *J. Biol. Chem.* 282 33275–33283. 10.1074/jbc.M702800200 17827157

[B10] Clinical and Laboratory Standards Institute [CLSI] (2006). *Performance Standards for Antimicrobial Susceptibility Testing; Sixteenth Informational Supplement Document M100-S16.* Wayne, PA: CLSI.

[B11] Clinical and Laboratory Standards Institute [CLSI] (2015). *Performance Standards for Antimicrobial Susceptibility Testing; Twenty-Fifth Informational Supplement Document M100-S25.* Wayne, PA: CLSI.

[B12] Congrui Z. Mingxu Z. Jie T. Guoqiang Z. (2013). Cloning and sequencing of housekeeping gene gapA from different originated *Escherichia coli*. *China Poult.* 35 21–23. 10.3969/j.issn.1004-6364.2013.17.007

[B13] Dang H. Lovell C. R. (2016). Microbial surface colonization and biofilm development in marine environments. *Microbiol. Mol. Biol. Rev.* 80 91–138. 10.1128/MMBR.00037-15 26700108 PMC4711185

[B14] Farkas A. Craciunas C. Chiriac C. Szekeres E. Coman C. Butiuc-Keul A. (2016). Exploring the role of coliform bacteria in class 1 integron carriage and biofilm formation during drinking water treatment. *Microb. Ecol.* 72 773–782. 10.1007/s00248-016-0758-0 27079455

[B15] González Barrios A. F. Zuo R. Hashimoto Y. Yang L. Bentley W. E. Wood T. K. (2006). Autoinducer 2 controls biofilm formation in *Escherichia coli* through a novel motility quorum-sensing regulator (MqsR, B3022). *J. Bacteriol.* 188 305–316. 10.1128/JB.188.1.305-316.2006 16352847 PMC1317603

[B16] Ito A. Taniuchi A. May T. Kawata K. Okabe S. (2009). Increased antibiotic resistance of *Escherichia coli* in mature biofilms. *Appl. Environ. Microbiol.* 75 4093–4100. 10.1128/AEM.02949-08 19376922 PMC2698376

[B17] Juhna T. Birzniece D. Rubulis J. (2007). Effect of phosphorus on survival of *Escherichia coli* in drinking water biofilms. *Appl. Environ. Microbiol.* 73 3755–3758. 10.1128/AEM.00313-07 17416695 PMC1932671

[B18] Lee J. Shin S. Teng C.-H. Hong S. J. Kim K. S. (2005). FimH adhesin of *Escherichia coli* K1 type 1 fimbriae activates BV-2 microglia. *Biochem. Biophys. Res. Commun.* 334 917–923. 10.1016/j.bbrc.2005.06.180 16036224

[B19] Liu X. Matsumura P. (1994). The FlhD/FlhC complex, a transcriptional activator of the *Escherichia coli* flagellar class II operons. *J. Bacteriol.* 176 7345–7351. 10.1128/jb.176.23.7345-7351.1994 7961507 PMC197124

[B20] Livak K. J. Schmittgen T. D. (2001). Analysis of relative gene expression data using real-time quantitative PCR and the 2^-ΔΔC_T_^ method. *Methods* 25 402–408. 10.1006/meth.2001.1262 11846609

[B21] Maeda H. Fujimoto C. Haruki Y. Maeda T. Kokeguchi S. Petelin M. (2003). Quantitative real-time PCR using TaqMan and SYBR Green for *Actinobacillus actinomycetemcomitans, Porphyromonas gingivalis, Prevotella intermedia, tetQ* gene and total bacteria. *FEMS Immunol. Med. Microbiol.* 39 81–86. 10.1016/S0928-8244(03)00224-4 14557000

[B22] Moreira J. M. R. Gomes L. C. Araújo J. D. P. Miranda J. M. Simões M. Melo L. F. (2013). The effect of glucose concentration and shaking conditions on *Escherichia coli* biofilm formation in microtiter plates. *Chem. Eng. Sci.* 94 192–199. 10.1016/j.ces.2013.02.045

[B23] Niu C. Robbins C. M. Pittman K. J. Osborn J. L. Stubblefield B. A. Simmons R. B. (2013). LuxS influences *Escherichia coli* biofilm formation through autoinducer-2-dependent and autoinducer-2-independent modalities. *FEMS Microbiol. Ecol.* 83 778–791. 10.1111/1574-6941.12034 23078586

[B24] Pratt L. A. Kolter R. (1998). Genetic analysis of *Escherichia coli* biofilm formation: roles of flagella, motility, chemotaxis and type I pili. *Mol. Microbiol.* 30 285–293. 10.1046/j.1365-2958.1998.01061.x 9791174

[B25] Rochelle-Newall E. Nguyen T. M. H. Le T. P. Q. Sengtaheuanghoung O. Ribolzi O. (2015). A short review of fecal indicator bacteria in tropical aquatic ecosystems: knowledge gaps and future directions. *Front. Microbiol.* 6:308. 10.3389/fmicb.2015.00308 25941519 PMC4400915

[B26] Romling U. Balsalobre C. (2012). Biofilm infections, their resilience to therapy and innovative treatment strategies. *J. Intern. Med.* 272 541–561. 10.1111/joim.12004 23025745

[B27] Sanchez-Vizuete P. Orgaz B. Aymerich S. Le Coq D. Briandet R. (2015). Pathogens protection against the action of disinfectants in multispecies biofilms. *Front. Microbiol.* 6:705. 10.3389/fmicb.2015.00705 26236291 PMC4500986

[B28] Shrestha S. R. Afsar A. (2017). Mutation in *flrA* and *mshA* genes of *Vibrio cholerae* inversely involved in *vps*-independent biofilm driving bacterium toward nutrients in lake water. *Front. Microbiol.* 8:1770. 10.3389/fmicb.2017.01770 28959249 PMC5604084

[B29] Soto S. M. (2013). Role of efflux pumps in the antibiotic resistance of bacteria embedded in a biofilm. *Virulence* 4 223–229. 10.4161/viru.23724 23380871 PMC3711980

[B30] Taub F. B. (1997). Unique information contributed by multispecies systems: examples from the standardized aquatic microcosm. *Ecol. Appl.* 7 1103–1110. 10.1890/1051-0761(1997)007[1103:UICBMS]2.0.CO;2

[B31] Thevenon F. Adatte T. Wildi W. Pote J. (2012). Antibiotic resistant bacteria/genes dissemination in lacustrine sediments highly increased following cultural eutrophication of Lake Geneva (Switzerland). *Chemosphere* 86 468–476. 10.1016/j.chemosphere.2011.09.048 22051343

[B32] Xiao Z. Yu D. Ali L. Lin J. Wang Y. Zhang J. (2015). Glucose-induced resistance to ciprofloxacin and erythromycin in Enterococci. *Enliven J. Anesthesiol. Crit. Care Med.* 2 24–29. 10.1016/j.chemosphere.2012.04.014 22633859

[B33] Yu D. Yi X. Ma Y. Yin B. Zhuo H. Li J. (2009). Effects of administration mode of antibiotics on antibiotic resistance of *Enterococcus faecalis* in aquatic ecosystems. *Chemosphere* 76 915–920. 10.1016/j.chemosphere.2009.04.057 19476969

[B34] Yu D. J. Lai B. S. Li J. Ma Y. F. Yang F. Li Z. (2012). Cornmeal-induced resistance to ciprofloxacin and erythromycin in enterococci. *Chemosphere* 89 70–75. 10.1016/j.chemosphere.2012.04.014 22633859

[B35] Zhang J. Wang Y.-Q. Li J. Ma Y.-F. Lin J. Xiao Z.-R. (2016). Phenotypic and genotypic analysis of vancomycin-resistant Enterococci strains isolated from different water sources. *Afr. J. Bacteriol. Res.* 8 8–13.

[B36] Zippel B. Rijstenbil J. Neu T. R. (2007). A flow-lane incubator for studying freshwater and marine phototrophic biofilms. *J. Microbiol. Methods* 70 336–345. 10.1016/j.mimet.2007.05.013 17590463

