# Supplementary figures and images for: Biofilm Formation Plays a Role in the Formation of Multidrug-Resistant Escherichia coli Toward Nutrients in Microcosm Experiments
Source: Front Microbiol. 2018 Mar 2;9:367. doi: 10.3389/fmicb.2018.00367 (PMC5840168; doi:10.3389/fmicb.2018.00367)

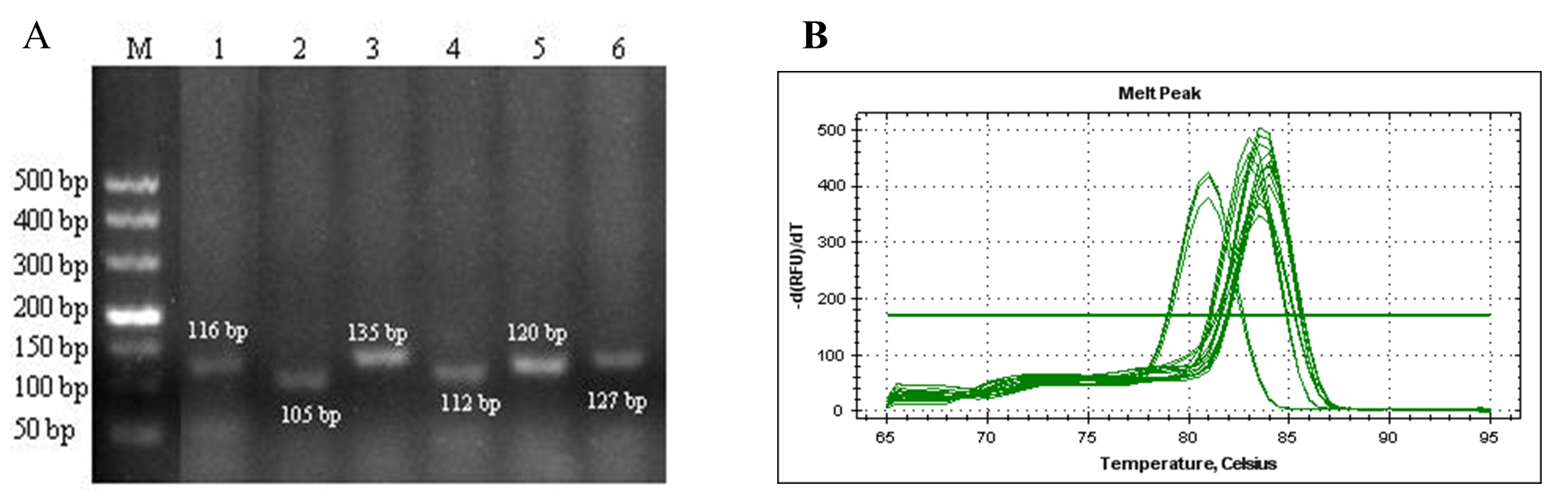

Supplement: FIGURE S1 — (A) Electrophoresis result of six targeted genes “M” is DL500 marker; 1, 2, 3, 4, 5 and 6 is luxS, mqsR, flhD, fliA, motA, and fimH PCR products, respectively. (B) Melting peak of luxS, mqsR, flhD, fliA, motA, and fimH gene. [file Image_1.TIF]

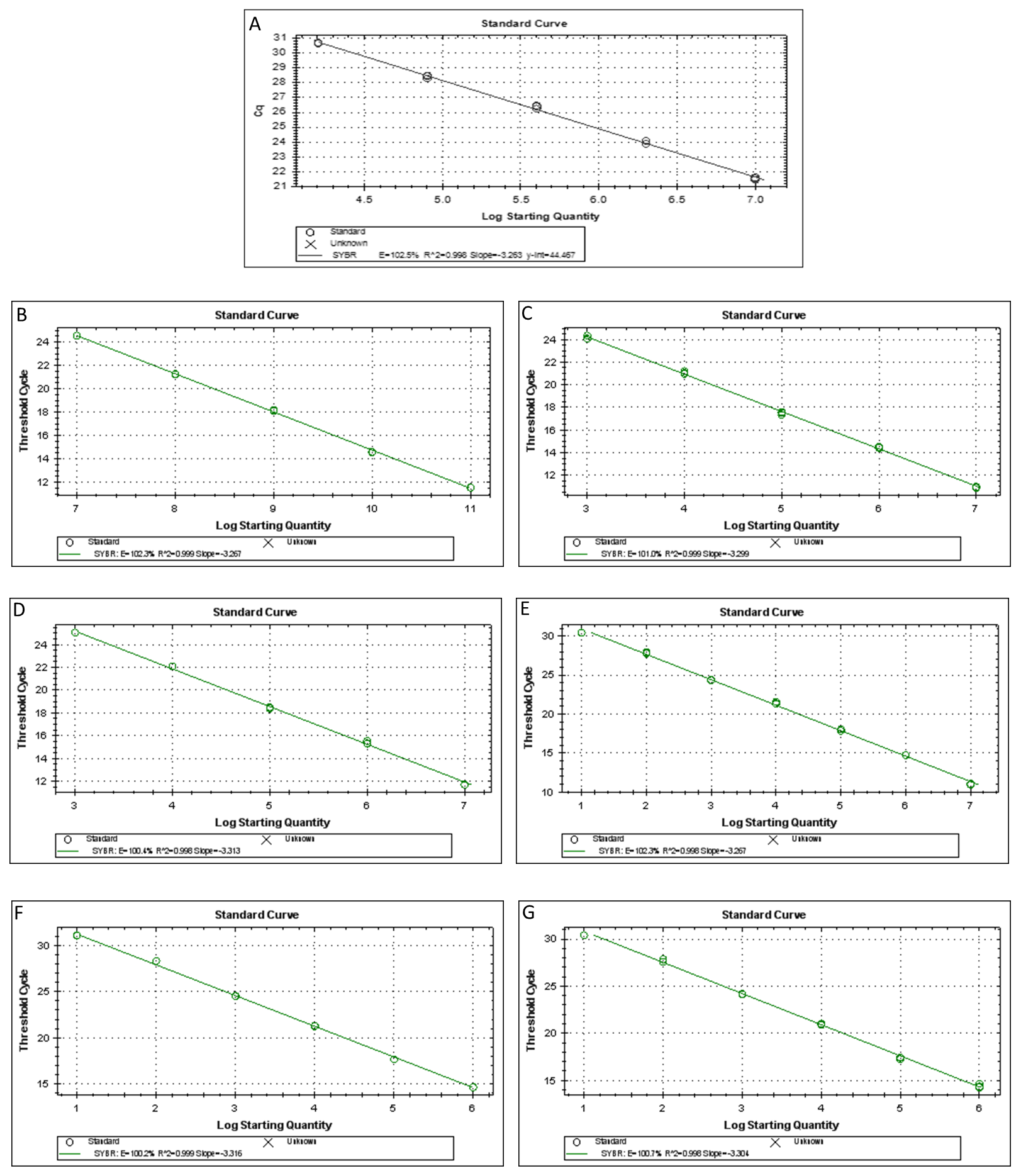

Supplement: FIGURE S2 — Standard curve of (A) gapA, (B) luxS, (C) mqsR, (D) flhD, (E) fliA, (F) motA, and (G) fimH gene. [file Image_2.TIF]

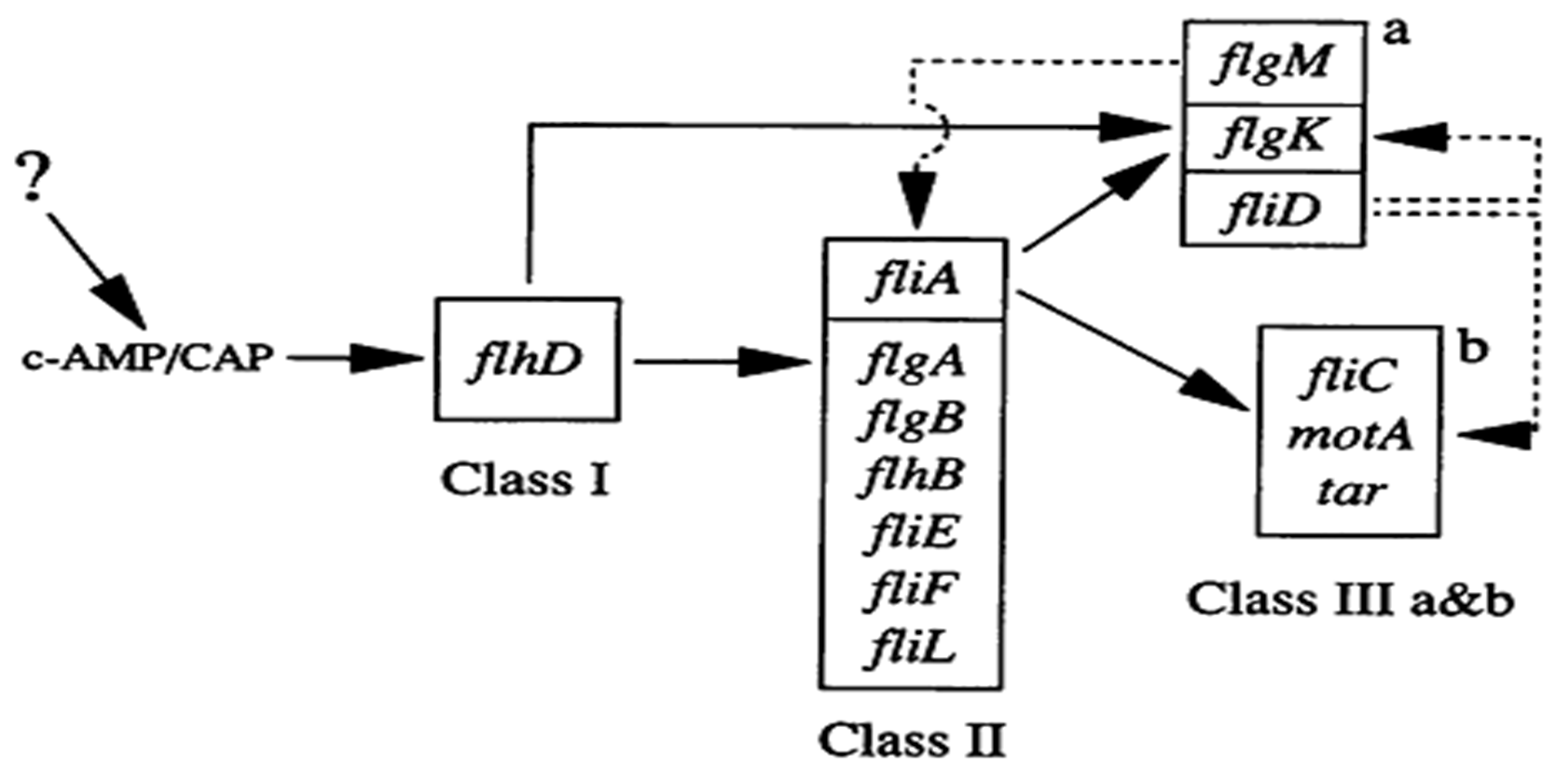

Supplement: FIGURE S3 — The relationships between biofilm-forming genes are used with permission of (Liu and Matsumura, 1994). [file Image_3.TIF]
